# Ambulance helicopter contribution to search and rescue in North Norway

**DOI:** 10.1186/s13049-016-0302-8

**Published:** 2016-09-13

**Authors:** Ragnar Glomseth, Fritz I. Gulbrandsen, Knut Fredriksen

**Affiliations:** 1 Anaesthesia and Critical Care Research Group, Department of Clinical Medicine, Faculty of Health Sciences, UiT the Arctic University of Norway, N-9037 Tromsø, Norway; 2 The National Air Ambulance Service of Norway, POB 235, N-8001 Bodø, Norway; 3 The 330 Squadron, Royal Norwegian Air Force, Air wing 137, N-4050 Sola, Norway; 4 Division of Emergency Medical Services, University Hospital of North Norway, N-9038 Tromsø, Norway

**Keywords:** Search and rescue, Helicopter emergency medical service, Air ambulance

## Abstract

**Background:**

Search and rescue (SAR) operations constitute a significant proportion of Norwegian ambulance helicopter missions, and they may limit the service’s capacity for medical operations. We compared the relative contribution of the different helicopter resources using a common definition of SAR-operation in order to investigate how the SAR workload had changed over the last years.

**Methods:**

We searched the mission databases at the relevant SAR and helicopter emergency medical service (HEMS) bases and the Joint Rescue Coordination Centre (North) for helicopter-supported SAR operations within the potential operation area of the Tromsø HEMS base in 2000–2010. We defined SAR operations as missions over land or sea within 10 nautical miles from the coast with an initial search phase, missions with use of rescue hoist or static rope, and avalanche operations.

**Results:**

There were 769 requests in 639 different SAR operations, and 600 missions were completed. The number increased during the study period, from 46 in 2000 to 77 in 2010. The Tromsø HEMS contributed with the highest number of missions and experienced the largest increase, from 10 % of the operations in 2000 to 50 % in 2010. Simple terrain and sea operations dominated, and avalanches accounted for as many as 12 % of all missions. The helicopter crews used static rope or rescue hoist in 141 operations.

**Discussion:**

We have described all helicopter supported SAR operations in our area by combining databases. The Tromsø HEMS service had taken over one half of the missions by 2010. Increased availability for SAR work is one potential explanation.

**Conclusions:**

The number of SAR missions increased during 2000-2010, and the Tromsø HEMS experienced the greatest increase in workload.

## Background

The National Air Ambulance Service of Norway is more involved in SAR operations than other Scandinavian services [1, 2], and it has experienced an increasing number of SAR requests [3, 4]. In contrast to healthcare services, Norwegian SAR is organised by the police authorities, and the Air Force’s 330 Squadron provides dedicated helicopter support for SAR [5]. However, the services overlap, and both contribute to both SAR and ambulance missions.

The University Hospital of North Norway (UNN) HEMS base (Tromsø HEMS) is located midway between two SAR bases, and has seen an increase in SAR missions [4]. The Tromsø HEMS fills a geographic gap in the SAR coverage, and has become increasingly capable of solving SAR requests, including static rope operations to sites where landing the aircraft is not possible. However, the demand for ambulance operations is also increasing, and a number of requests are declined because of the total workload of the service [4].

The distinction between SAR and air ambulance missions is not always clear, as ambulance missions in our area often include elements of both search and rescue before medical treatment is possible, and many SAR missions involve persons with trauma or medical problems. We collected data about helicopter based SAR from all relevant services in the area where the Tromsø HEMS is a potential resource. A definition of SAR common to all services was used to allow comparison of the available helicopter resources’ relative contribution, and to describe the nature of region’s SAR operations. Our hypothesis was that HEMS is involved in an increasing proportion of the SAR operations.

## Methods

### Study design

The study was a retrospective cohort study of all SAR missions requested in the extended catchment area of the Tromsø HEMS during 2000–2010.

### Setting and geographical definitions

**Fig. 1:**
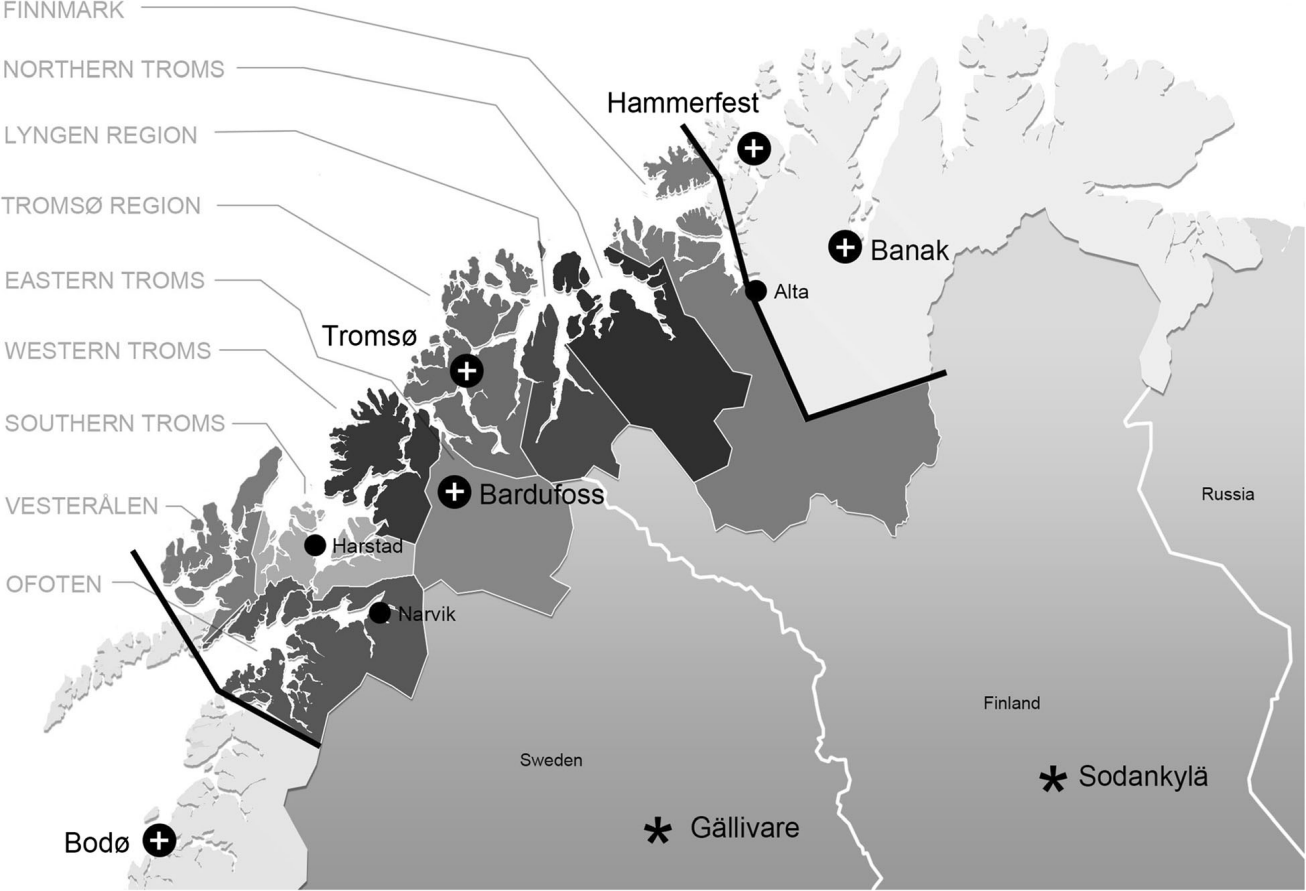
Map of North Norway and neighbour regions of Sweden, Finland and Russia. The limits of the study area is marked with thick black lines. Helicopter bases are indicated as +, and the geographic regions referred in the text are indicated. The closest HEMS bases in Finland and Sweden are indicated as *

In a narrow sense, the Tromsø HEMS catchment area is the same as for the UNN hospital trust (Fig. 1), covering a population of 183 500 and an area of 30 000 sq.km. The area is sparsely populated outside the cities, with an average population density in the two northernmost counties of 2 and 6 inhabitants per sq.km [6]. The service operates occasionally also in the neighbouring regions of Norway, Sweden and Finland. We included all SAR operations in this *extended area*, and divided the area in smaller geographical *regions* (shown in Fig. 1).

Table 1 shows the helicopter resources available for SAR in the area, and Fig. 1 shows the localisation of the bases, including the nearest resources in Sweden and Finland. The Emergency Medical Communication Centre (EMCC) at the UNN Tromsø coordinates and dispatches the ambulance missions of Tromsø HEMS, and the Joint Rescue Coordination Centre (JRCC) in Bodø coordinates all SAR missions [5].

**Table 1:** Overview of helicopter resources available for search and rescue operations in the study area

Resource	Base	Owner	Helicopter	Crew	Capacity	Max speed (km/h)	Flight endurance	Hoist/ SR	Function
Tromsø HEMS	Tromsø	LAT ANS	Agusta Westland AW 139	Pilot, Doc, RS.	10	306	5 h	SR	HEMS / Ambulance
330 Banak	Banak	RNoAF 330sq	Westland Sea King	Pilot *x*2, Navigator, Mechanic, Doc, RS.	13	230	5,5 h	Hoist	SAR
330 Bodø	Bodø	RNoAF 330sq	Westland Sea King	Pilot *x*2, Navigator, Mechanic, Doc, RS.	13	230	5,5 h	Hoist	SAR
339 Sq	Bardufoss	RNoAF 339sq	Bell 412	Pilot, Navigator	13	259	3,5 h		Military transport
337 Sq	Coastguard ships	RNoAF 337sq	Westland Lynx	Pilot, Navigator, Mechanic, RS	10	305	4 h	Hoist	SAR, Fishery surveillance
Hammerfest	Hammerfest	Statoil	Eurocopter EC 225	Pilot *x*2, Mechanic, Doc, RS.	15	270	5 h	Hoist	SAR resource petroleum industry

Capacity: maximum number of persons carried. *HEMS* helicopter emergency medical service, *SR* static rope, *LAT ANS* The National Air Ambulance Service of Norway, *Doc* doctor, *RS* rescue-swimmer or rescue-man, *Sq* squadron, *SAR* search and rescue

### Data sources, variables and definitions

We used the following terms consistently: A SAR *operation* is an event that calls for coordination of available SAR resources. One SAR operation may lead to a *request* for assistance from one or more helicopter resources. A request may in turn lead to a SAR *mission*, and thus a SAR operation may consist of more than one mission.

We classified operations in *categories*: *sea* (inside 10 nM from the coastline), *simple terrain* (flat terrain, no belaying necessary), *demanding terrain* (evacuating an immobilised patient will require belaying, normal movement possible without belaying), *alpine terrain* (steep terrain, all movements requires belaying, may require use of climbers), or *avalanches*.

We searched the databases of the 330 sq., the Tromsø HEMS, and the JRCC for SAR requests to locations within the study area from 2000 until the end of 2010. We recorded time data, SAR location and category, and the requested helicopter resource. When necessary, we consulted the actual mission crews for details.

We applied a common definition of a SAR mission: i.e. missions that included at least one of the following: unknown localisation of the casualty necessitating an initial search phase, use of rescue hoist or static rope, or avalanches. We limited the inclusion of missions over sea to operations within 10 nM from the Norwegian coastline, as only these are relevant for the Tromsø HEMS. This excluded far sea-operations common to the 337, 330 and the Hammerfest helicopter. We excluded secondary searches for assumed dead persons, but included a second search when based on new information that increased the possibility to find the casualty alive.

### Statistical analysis

A linear regression line was calculated to visualise the change over time for selected parameters, using Microsoft Excel® 2008 software.

### Approval

The Hospital’s Data Protection Officer approved the study as a quality improvement project (2012/412, 31.01.2012).

## Results

The six helicopter resources were requested 769 times for 639 SAR operations, which means that more than one resource was requested for a number of missions. 600 out of the 769 requests resulted in completed missions.

**Fig. 2:**
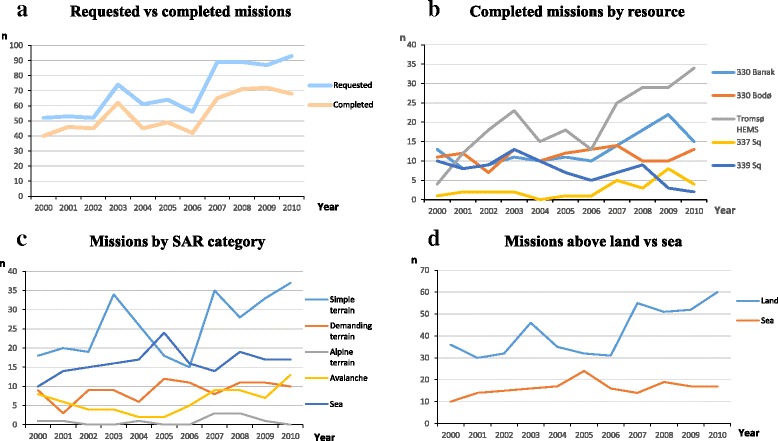
The annual number of search and rescue (SAR) missions for: **a** Requested and completed missions for all helicopter resources together. **b** SAR missions over the study period for the individual helicopter resources. The Hammerfest helicopter is omitted from the figure as it contributed with only one mission in 2000 and two in 2008. **c** SAR missions per year sorted by SAR category. **d** All SAR missions divided in missions above sea and land

The number of operations increased from 46 in 2000 to 77 in 2010 (Fig. 2a). Figure 2b shows the annual contribution of the individual helicopter resources. The most striking finding was that the Tromsø HEMS increased its contribution from 10 to 50 % of all SAR missions during the study period and accounts for most of the total increase. In addition, the 330 sq. at Banak and the 337 sq. experienced a small increase, but the other resources did not have any increase. Approximately 25 % of the requested missions were not completed, because of either weather conditions, technical problems, duty time regulations, or a concurrent operation.

Operations into simple terrain dominated, accounting for more operations than the sea-operations in all years but two (Fig. 2c). The Tromsø HEMS and 330 sq. spend much time preparing for mountain rescue, but the categories “alpine” and “demanding terrain” comprised no more than ca 10 operations/year. Alpine rescue was uncommon (0–3/year). On the other side, avalanche rescue was prevalent (8–13/year) and increasing. The number of sea operations was also increasing, but less than the other categories (Fig. 2d).

**Fig. 3:**
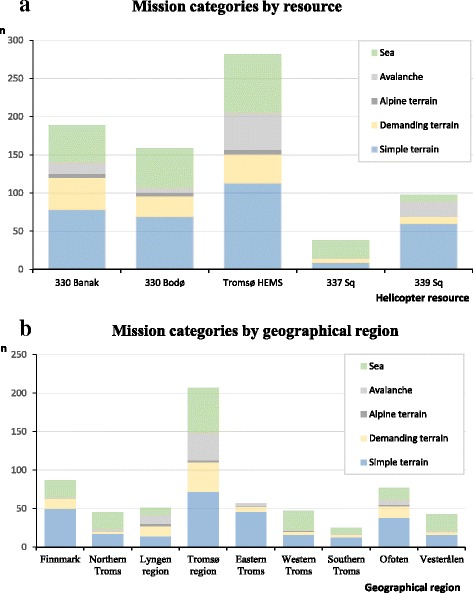
**a** The search and rescue (SAR) missions broken down by mission categories and shown for the individual helicopter resources, except the for the Hammerfest helicopter that contributed with only three missions during the study period. **b** The distribution of SAR categories in the geographical regions in the study area

Because the primary responsibilities of the resources differ, we analysed the relative distribution of SAR categories among the resources (Fig. 3a). The dominating category for the coast guard’s 337 sq. was sea rescue, and the dedicated SAR resource at sea, the 330 sq., did 25–30 % sea rescue, even though our study did not include operations outside the coastal waters. Simple terrain rescue dominated for all resources except the 337 sq. Missions into demanding or alpine terrain were slightly more frequent in the 330 helicopters, but also comprised 14 % of the SAR workload for the Tromsø HEMS. Interestingly, avalanche rescue was prevalent and represented 12 % of all SAR missions, and as much as 17 % of the Tromsø HEMS missions. The total number of SAR missions were much lower for the 337 and 339 sq. than the 330 and HEMS bases, and the Hammerfest helicopter contributed with only three sea-operations.

More than 200 of the 639 operations took place in the densely populated Tromsø region. In addition, the alpine Lyngen and the Eastern Troms had an increasing number of missions during the period. We analysed the prevalence of SAR categories by geographical region (Fig. 3b), and operations in demanding terrain were most frequent in the Lyngen, Tromsø and Ofoten regions (19–25 %). Alpine operations were uncommon, except in Lyngen (6 %). Avalanches constituted a significant part of the SAR operations in Lyngen (24 %) and Tromsø (17 %), but only 2–9 % in the other regions. Simple terrain was the most common SAR category in most regions, and accounted for as much as 81 % of all operations in Eastern Troms. The exceptions were sea rescue (18–53 %) which was the most prevalent category in Northern and Western Troms and in the Vesterålen area.

**Fig. 4:**
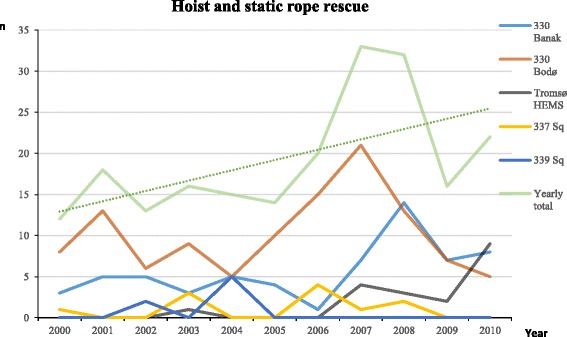
The number of persons rescued by hoist and static rope by the individual helicopter resources, and by all resources together, over the years 2000–2010. The Hammerfest helicopter rescued 26 persons in one single mission in 2000. This extraordinary mission was not included in the figure or when the simple regression line was calculated, only to illustrate the general trend over the study period

Access to sites where the aircraft cannot land is important for all SAR categories, probably except for simple terrain. Most SAR services operate rescue hoists to meet this demand, but the Norwegian HEMS helicopters use a fixed length static rope. The services rescued 237 persons with hoist or static rope in 141 different operations, and the number of hoist/rope operations increased during the period (Fig. 4). An exception to this steady increase was one single operation in 2000 where 26 persons were hoisted from a wrecked ship, an extraordinary occasion that accounted for almost 10 % of all persons evacuated by hoist or rope during the entire period. We omitted this exceptional operation from in Fig. 4, only to emphasise the general trend.

Figure 4 demonstrates the individual resources’ contribution to hoist and rope operations over the years. The two dedicated SAR helicopter resources contributed to the majority of these operations hoisting between 1 and 20 persons/year. The Tromsø HEMS started regular static rope missions as late as 2007, and the number of static rope rescue missions have increased after this.

**Fig. 5:**
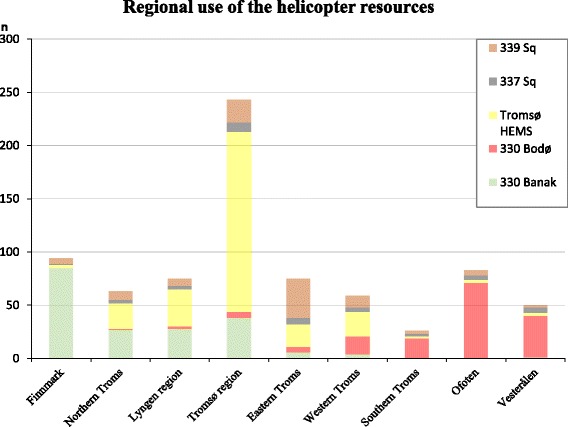
The individual helicopter resources contribution to search and rescue missions in the different geographical regions of the study area

The regional differences in helicopter resource usage showed that the nearest resource was preferred, followed by the other resources according to their distance from the region (Fig. 5).

## Discussion

We found 769 requests for helicopter assistance in 639 during the 11 year study period. The number of missions increased from 46 to 77 over the years, and the Tromsø HEMS had the highest mission number and the largest increase. Simple terrain and sea operations dominated, but avalanches accounted for as many as 12 % of all missions.

Different SAR definitions have made it impossible to compare the contribution of different SAR resources. The distinction between HEMS and SAR operations is not clear, and many rescued victims are in need of medical treatment. Furthermore, ambulance patients may need evacuation from scenes that are not readily accessible to ground ambulance personnel. For this reason, we conceived the present study, and collected data from all resources using a common definition of SAR.

One interesting finding is the magnitude of helicopter-based SAR in North Norway. The study excluded operations outside 10 nM from the coastline, and comprised an area with only approximately 250 000 inhabitants. However, the vast geographic area and low population density in the northernmost counties of Norway (2–6 inhabitants per sq km^2^) may at least in part explain the need for helicopter support in many SAR operations [6]. Indeed, the population is covered by a relatively large number of helicopter resources, in order to compensate for the vast areas and the long distances between hospitals.

As described, we excluded the far sea operations, to make the inclusion relevant for the Tromsø HEMS. It was our intention to describe the relative contribution of the available resources in a geographical region where an important ambulance resource, the HEMS, had experienced an increase in SAR requests. We found that the Tromsø region had more SAR operations than the other regions in the study area, and the Tromsø HEMS increased its contribution from 10 to 50 % during the period. This is understandable, at least in part, since we defined the study area as the potential area of the HEMS.

One potential problem with the increased SAR work for the Tromsø HEMS is that the total number of missions is close to the limits of the service. During 2008–2011, the service cancelled 13–18 missions/year because of duty time, and approximately 30 missions/year because of concurring requests [4]. The numbers are small, but they were close to zero before 2000. Even though SAR still comprises only ca 5 % of the Tromsø HEMS missions, and medical missions constitute most of the increased workload, we expect that the service will not be able to handle a further development in SAR operations, in line with the changes we show in this study. In addition, the regional health trust depends on an effective air ambulance service to ensure optimal patient flow between hospitals. In this perspective, an uncontrolled increase of non-healthcare operations is alarming.

Our study does not answer how increased availability of the Tromsø HEMS has driven the observed development. Still, it is a fact that Tromsø HEMS started regular static rope missions during the study period, and has focused both training and equipment increasingly more on SAR operations. The resource has thus become an important supplement for SAR operations, and the services proximity to common accident scenes in the Tromsø and Lyngen regions is important, as the dedicated SAR helicopters are based more than one hour away. Thus, the HEMS may respond faster, use less mission time at lower costs than other resources. In addition, only a limited number of the operations demand the specialised competencies of the 330 sq. crews.

The helicopters are frequently used for simple terrain operations, and we believe that few of these operations are urgent from a medical point of view. It is known from the 330 sq. offshore operations that a significant amount of the patients are hospitalised and some may probably not have survived without early medical treatment [7]. Furthermore, studies from mountain-near services in Central Europe have shown a high number of alpine sports injuries that require advanced on scene medical treatment [8–10]. We believe that the operations described in the present study may differ from the off shore operations, and from the HEMS in busy alpine sport resorts in this respect. However, we did not study how often the competence of the physician on board was used, and we suggest that a subsequent study evaluates this. Another intriguing question is that many operations could have been solved without helicopter, particularly among the simple terrain operations.

On the contrary, it is evident that the helicopter is beneficial if a victim needs evacuation by hoist or static rope. We have described that the number of hoist and rope operations was increasing during the study period. The Tromsø HEMS has a long tradition in landing “light on wheels” with the rotor running in sloping terrain, and solved the majority of terrain missions by this simple operation. This is in contrast to other countries where such evacuations would mandate a rope or hoist rescue [11]. For this reason, we were not surprised to find that all the Tromsø HEMS static rope operations were in demanding or alpine terrain, where the helicopter often was necessary for the evacuation. The ability to work in sloping terrain even without hoist or static rope in the majority of these missions may have lowered the threshold to request the Tromsø HEMS for terrain operations.

Alpine skiing has become increasingly popular in the region over the latest decades, and accidents from this activity accounts for some of the observed increase in demanding and alpine terrain operations, particularly in the Tromsø and Lyngen regions. Also important, avalanches represent as much as 17 and 24 % of the operations in Tromsø and Lyngen, respectively, and the Tromsø HEMS may reach these regions within 10–15 min. Avalanches are particularly demanding with respect to access time and crew training [12–14]. However, Tromsø HEMS has specialised on rapid avalanche rescue within the narrow time frame of avalanche survival, and this is obviously an important use of the HEMS resource.

A consensus report for mountain rescue emphasizes the need for rapid dispatch and integration into local EMS systems to secure a smooth transition from the pre-hospital environment to advanced hospital treatment [15]. This suggestion is in accordance with the Norwegian organisation, where the hospital trusts are responsible both for the EMCC and the medical staffing of the HEMS. However, the report recommends access to the scene within 20 min and a maximum service diameter for the helicopter bases of 50 km, which is based on central European geography and infrastructure, and does not make sense in the thinly populated North. On the other hand, access to the hospital based EMCC via the emergency medical telephone number 113 is well implemented in Norway and ensures robust integration and coordination of medical resources. The EMCC-coordinated co-operation of pre- and intra-hospital resources has been demonstrated on several occasions in Tromsø, especially for avalanche victims and patients with severe accidental hypothermia [16, 17]. The Norwegian system also provides immediate integration with other emergency institutions, like the police, fire brigade and JRCC.

Since the medical staffing in the 330 helicopters and the Tromsø HEMS is almost identical, the resources may supplement each other. E.g., the services operates with Rescue-man/ Paramedic and HEMS physicians that fulfil national minimum standards [18]. Still, the services are not completely interchangeable. The HEMS is part of the health trust services, and the JRCC controls the 330 sq. resources.

It is possible that insufficient capacity of dedicated SAR helicopters of the 330 sq. shifted some of the total workload to Tromsø HEMS. Indeed, a high number of SAR requests originate midway between the 330 sq. bases at Banak and in Bodø, and the results of the present study may support this notion. In addition, the old 330 sq. Sea-King aircrafts have experienced maintenance problems for years, and they will be replaced. This may also have reduced the capacity of the dedicated SAR resource, and increased the SAR workload for HEMS.

The availability of the 339 sq. helicopters was reduced as the squadron was involved in the Norwegian military aeromedical detachment in Afghanistan from 2008, probably reflected in Fig. 2b. The future of this squadron is also unclear, and it has been suggested to move the resource to Southern Norway in close future. On the other side, HEMS availability has increased in the region, as a new HEMS base was established after the study period (2015) at Evenes, on the border between the Southern Troms and Ofoten regions. It is still not clear how this respurce will influence SAR operations in the region.

Still, there are several important questions that remain: To what extent does increased availability for SAR operations of the Tromsø HEMS contribute to the figures? How many operations could have been solved without helicopter? We also need to know more about the need for medical interventions during our SAR missions, as this could point to more use of non-healthcare resources. Though the number of missions is not directly related to the financial costs of running the air ambulance services like HEMS bases, it is plausible that increased HEMS use will lead to a demand for new bases or helicopters in the future.

Answers to these questions are important both for the future structure of health care services in the region, for the future distribution of HEMS bases, and to decide whether the Tromsø area should have a dedicated SAR helicopter service, or whether the “SAR light” operations of the HEMS should be developed further and expanded.

## Conclusions

Our findings support the hypothesis that the Tromsø HEMS service experienced the greatest increase in SAR operations in 2000–2010, and the service performed half of the operations by the end of the study period. Increased availability for SAR work, especially the capability of static rope operations, is one potential explanation. Long distances to the nearest designated SAR helicopters is another probable reason.

## Abbreviations

EMCC: Emergency Medical Communication Centre
HEMS: Helicopter emergency medical service
JRCC: Joint Rescue Coordination Centre
SAR: Search and rescue
UNN: University Hospital of North Norway
